# A Robust Normally Closed Pneumatic Valve for Integrated Microfluidic Flow Control

**DOI:** 10.3390/mi16010034

**Published:** 2024-12-29

**Authors:** Minggan Li, Siqin Dong

**Affiliations:** Zepto Life Technology Inc., 1000 Westgate Drive, St. Paul, MN 55114, USA

**Keywords:** microfluidic, lab on a chip, normally closed valve, in vitro diagnostic (IVD)

## Abstract

Accurate fluid management in microfluidic-based point-of-care testing (POCT) devices is critical. Fluids must be gated and directed in precise sequences to facilitate desired biochemical reactions and signal detection. Pneumatic valves are widely utilized for fluid gating due to their flexibility and simplicity. However, the development of reliable normally closed pneumatic valves remains challenging, despite their increasing demand in advanced POCT applications to prevent uncontrolled fluid flow. Existing normally closed valves often suffer from poor reliability and lack precise control over fluid opening pressure, due to the uncontrolled stretching of the elastomer during assembly. In this study, we propose and develop a robust method for normally closed valves. By precisely controlling the pre-stretching of the elastomer, we achieve reliable valve closure and accurate control of the opening pressure. A robust normally closed valve was designed and fabricated, and its pneumatic opening pressure was systematically studied. Experimental validations were conducted to demonstrate the reliability and effectiveness of the proposed design.

## 1. Introduction

Microfluidics has been widely adopted in the in vitro diagnostics (IVD) industry to integrate complex laboratory sample processing procedures and miniaturize large heavy testing equipment. These advancements have led to the development of numerous point-of-care testing (POCT) devices and their corresponding microfluidic disposable cartridges. Many of these devices, such as the Cepheid GeneXpert, Biocartis Idylla, Atlas Genetics, Alere, and Qiagen QiaStat-Dx, have established well-known performance benchmarks. Within each of these microfluidic cartridges, miniaturized valves are crucial for meeting the complex fluid flow control requirements. These valves ensure that fluids, such as samples and liquid reagents, are accurately directed to the correct reactions in the proper sequence, enabling precise and reliable testing results.

Fluid valving functions can be achieved through various methods, and different types of valves have been developed based on their operating principles. Examples include pinch valves [1,2], capillary valves [3,4], slip-chip valves [5], phase-change valves [6,7,8], rotary valves [9,10], and pneumatic valves [11,12,13,14,15], among others. While these valves have been employed in a range of applications, pneumatic valves have gained extensive use in microfluidic systems due to their design flexibility and simplicity in development. A typical pneumatic microfluidic valve consists of three layers: a pneumatic layer, a fluidic layer, and an elastomer film layer. The elastomer layer is sandwiched between the pneumatic and fluidic layers, allowing air pressure supplied from the pneumatic layer to deflect the elastomer and open or block a fluidic channel or port on the fluidic layer. This mechanism enables air pressure to control fluid flow within the fluidic layer by applying either positive or negative pressure. Furthermore, multiple pneumatic valves can be readily integrated into a single microfluidic device using a pneumatic manifold, enabling precise and programmable control in a desired sequence to meet sample processing requirements. As a result, pneumatic valves have been widely and successfully applied in various academic research studies [16,17,18] and commercial IVD products [19,20,21].

The aforementioned microfluidic pneumatic valves are typically normally open valves (NOVs), meaning that the fluid inside the device can start to flow before the valve is actuated and closed. This type of valve can be constructed by simply clamping an elastomer membrane between the fluidic and pneumatic layers without tightly blocking the flow paths. NOVs are suitable for devices where the fluid pressure in the flow path is minimal, and the liquid cannot flow freely under its own gravity. For example, in many lateral flow microfluidic applications, fluid volumes are often very small, sometimes as low as 20 µL, and typically cannot generate sufficient pressure to overcome the capillary forces of the microfluidic channels. Thus, it is safe to use normally open valves in such applications. However, with the emergence of many new diagnostic technologies, processing larger fluid volumes has become increasingly necessary. Liquid biopsy is one such example, often using body fluids like blood, plasma, or urine to identify diseases. In liquid biopsy applications, the biomarker concentrations are often extremely low, requiring a relatively large fluid volume, typically on the milliliter scale. For instance, Roche cobas and Biocartis Idylla’s lung cancer epidermal growth factor receptor (EGFR) mutation tests both use 2 mL of blood plasma as a specimen [22,23]. After mixing with lysis reagents, the total liquid volume can increase to 10 mL or more. When such large volumes are loaded into a microfluidic device, they generate significant head pressure within the channels. If an NOV is used in these cases, fluids may flow uncontrollably once the sample is loaded, as the fluid pressure overcomes the capillary forces of the microfluidic channel. A normally closed valve (NCV) addresses this issue by firmly blocking the fluidic channel initially, preventing uncontrolled flow. Consequently, NCVs are highly desirable for applications requiring large fluid volumes.

Recently, several designs for normally closed valves have been proposed [24,25,26], with efforts to control the fluid opening pressure [27]. However, due to the uncontrolled stretching of the elastomer during the pick-and-place assembly process, these valves often exhibit poor reliability in maintaining valve closure and limited controllability of the fluid opening pressure. In this article, we introduce a robust and fully controllable method for a normally closed pneumatic valve designed for microfluidic applications. This design allows the fluid opening pressure to be pre-determined and readily adjusted through precise elastomer stretching, offering a novel and reliable solution for microfluidic flow control.

## 2. Materials and Methods

**Figure 1:**
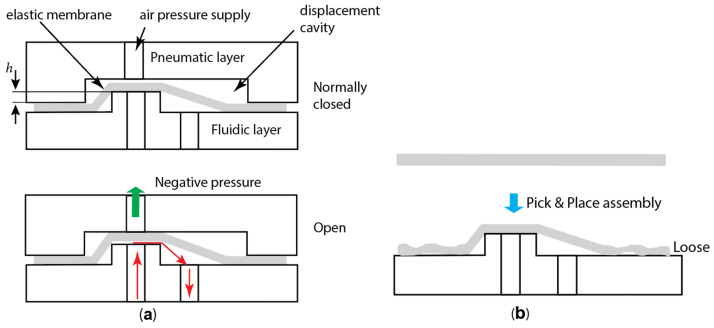
Schematic of an NCV and its assembly process: (**a**) schematic showing the NCV in its closed state (**top**) and open state (**bottom**). (**b**) Pick-and-place assembly process of the elastomer membrane.

A normally closed pneumatic microfluidic valve consists of a fluidic layer, a pneumatic control layer, and a flexible elastomer membrane, as schematically illustrated in Figure 1. A clamping bead surrounding the two fluidic vias compresses the membrane against the fluidic layer to create a gas-tight seal. An air pressure supply port in the pneumatic layer introduces negative pressure, deflecting the membrane upward into the displacement cavity. This deflection connects the two fluidic vias on the fluidic layer, allowing fluid to flow (Figure 1a). The valve is considered normally closed because one of the fluidic vias is raised, and the offset height *h* introduces a pre-load that prevents fluid flow. The opening pressure of the valve depends on this offset *h*. In theory, this valve design should function effectively. However, it lacks reliability and controllability for maintaining a normally closed state due to challenges in the pick-and-place membrane assembly process. During assembly, the membrane is placed onto the fluidic layer, and stretching of the membrane is expected due to the raised via. However, when the flexible thin membrane is laid onto the fluidic layer, it initially contacts the raised via. At this point, the membrane is not laterally constrained and naturally relaxes on the surface (Figure 1b). The clamping beads are then pressed onto the membrane to seal it. Consequently, the raised via cannot induce the necessary pre-stretch in the floppy membrane required to generate a controlled pressure to close the valve.

### 2.1. Principle of Normally Closed Valve Through Pre-Stretching

**Figure 2:**
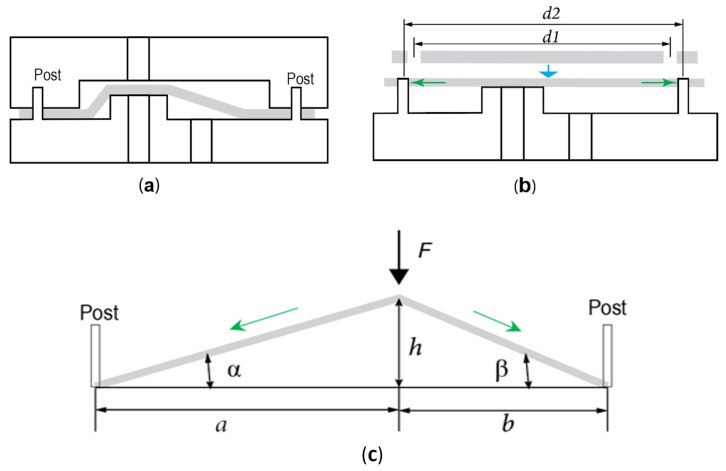
Schematic of a robust normally closed valve (NCV) and its assembly process. (**a**) Valve construction showing posts on the fluidic layer to constrain the elastomer membrane during assembly. (**b**) Membrane stretching determined by the offset between the posts and the holes on the membrane. (**c**) Schematic illustrating pressure generation through pre-stretch.

To achieve pre-stretch in the membrane during the assembly, we designed posts on the fluidic layer and corresponding holes on the membrane. The offsets between these posts and holes stretch the membrane during assembly, generating a pre-determined pressure on the raised via to close the valve (Figure 2a). The locations of the posts and holes can be strategically determined to generate the desired pre-stretch based on the fluid opening pressure requirements in the microfluidic system. For example, as shown in Figure 2b, if $d_{1}$ < $d_{2}$, the membrane will be pre-streched during assembly to place it onto the posts through those holes. Once assembly is complete and the membrane is clamped down, it will generate a pressure onto the raised via to close the valve. The greater the offset ($d_{1}$ − $d_{2}$), the higher the fluid opening pressure. Generally, a higher opening pressure requires a greater offset. Importantly, with this method, even if the pressure requirements change during product development, the valve design can be easily adjusted by modifying the distance of the holes, $d_{2}$, to match the updated opening pressure specifications. In practice, membranes and their corresponding holes can typically be laser-cut or die-cut, enabling rapid and cost-effective design changes. By inducing a pre-determined stretch in the membrane, this approach ensures the valve can reliably remain closed, while the fluid opening pressure is directly determined by the offset *d* between $d_{1}$ and $d_{2}$.

When using the valve in a microfluidic system, fluid fills the channel and reaches the via. The membrane over the via must generate sufficient pressure to keep the valve closed, preventing fluid flow under head pressure. Via membrane pressure (VMP) is the pressure exerted by the membrane on the via. A valve cannot remain normally closed if the head pressure of the fluid exceeds the VMP. Figure 2c shows a simplified one-dimensional schematic of pressure generation by pre-stretch. Consider that the two posts are the two vertices of a triangle at the bottom and the via is the top vertex. Then, the VMP can be obtained by calculating the compression force. Assume the distance from the raised via to the posts is *a* and *b*, respectively, the height of the raised via is *h*, and the strain of the membrane after stretching onto the post is ε; then, the compression force applied to the via *F* (Figure 2c) is

(1) $$F=Etw\varepsilon \left(\sin\alpha +\sin\beta \right),$$

where *E* is the Young’s modulus of the membrane, *t* is the thickness, *w* is the width of the membrane, and the strain $\varepsilon =\left(d_{2}-d_{1}\right)/\left(a+b\right)$. Thus, the VMP on the via can be obtained by dividing the compression force *F* by the via area. This simplified schematic demonstrates that the pressure is proportional to the strain or offset. If there is a higher head pressure, a greater offset is needed to ensure that the valve can be normally closed.

The vacuum opening pressure (VOP) is the vacuum supplied from the pneumatic layer to lift the membrane and open the valve.

Both the VMP and VOP can be numerically calculated. The calculated VMP determines whether the offset *d* is sufficient to stop fluid flow, thereby forming a normally closed valve. Meanwhile, the VOP provides guidance for selecting and regulating the vacuum source to ensure the valve can be successfully opened.

### 2.2. Normally Closed Valve Fabrication

**Figure 3:**
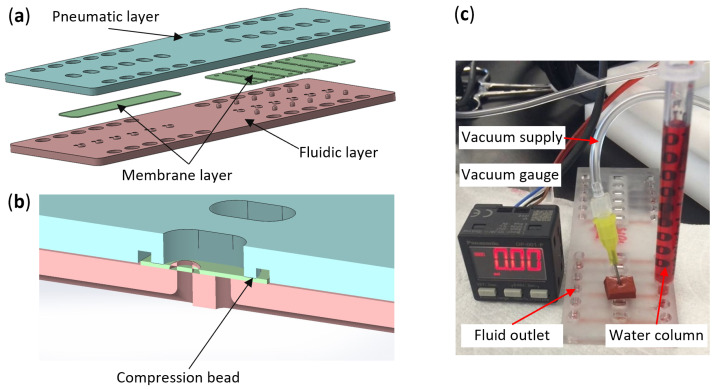
Normally closed valve design and test on a chip with different valve configurations. (**a**) Valve design with fluidic, pneumatic, and membrane layers. (**b**) Cross-section view of the valve construction. (**c**) Test setup.

To verify this concept, we designed a chip with pneumatic, fluidic, and elastomer membrane layers incorporating valve functions using SolidWorks 2020 (Figure 3a). In this chip, different raised via heights were incorporated: 0.381 mm, 0.406 mm, 0.432 mm, 0.448 mm, 0.477 mm, and 0.508 mm, respectively, to evaluate whether the valve could remain normally closed solely through the raised via (valve set A), as shown on the left half of the chip design in Figure 3a. On the same chip, a fixed via height of 0.508 mm was used, but posts were added on both sides of the vias to introduce pre-stretch in the membrane and assess the performance of the normally closed valve concept (valve set B), as shown on the right half of the chip design in Figure 3a. The chips were fabricated using a high-resolution 3D printing method (Protolabs, Maple Plain, MN), and the surface was treated to achieve a smooth finish.

The elastic membrane for the valves was laser cut using a BOSS Laser from a 0.25 mm thick BISCO liquid silicone rubber (LSR) sheet (Stockwell Elastomerics, Philadelphia, PA, USA) with a Shore A durometer of 40. For valve set A, a simple rectangular LSR membrane was cut, while for valve set B, two holes were added to enable assembly onto the posts. The distances between these two holes were varied to create different levels of pre-stretch in the membrane. The offset ($d_{2}$ − $d_{1}$) was adjusted to values of 0.1 mm, 0.2 mm, 0.3 mm, 0.4 mm, and 0.5 mm.

To assemble the valves, a 0.1 mm thick double-sided tape (PS-1340, Marian, Milwaukee, WI, USA) was used to bond the fluidic and pneumatic layers together. The tape was laser cut into specific shapes to avoid covering the elastic membrane area and was then bonded to the bottom side of the fluidic layer. After positioning the elastic membrane in the designated area (Figure 3a), the pneumatic layer was bonded to the top side of the tape, forming the testing valve device (Figure 3c). A total of three devices were fabricated.

### 2.3. Normally Closed Valve Test

To test the valves, a dyed water column was inserted into the hole on the right side of the valve, and a vacuum pump (KNF NMP830, KNF Neuberger Inc., Trenton, NJ, USA) was connected to the pressure supply hole in the center using a needle to provide negative pressure. This pump is capable of generating a negative pressure of −77,000 Pa (−11 psi gauge). A pressure gauge was connected to the pump line to measure the vacuum strength. If the valve opens, the dyed water flows through it and reaches the hole on the left side of the valve (Figure 3c).

Valve set A, which features various via heights, was tested to evaluate the valve’s ability to remain normally closed without applying positive pressure on the membrane to push it down. Different volumes of dyed water were loaded into a 1 mL syringe or a 10 mL pipette to generate water columns of 20 mm (196 Pa), 100 mm (980 Pa), and 200 mm (1961 Pa) in height, which were used to test the closing performance of each via height.

For valve set B, after calculating the VMP and VOP, a KNF vacuum pump capable of achieving a vacuum depth of −11 psi (gauge pressure) was selected to provide sufficient negative pressure to lift the membrane and open the valve. Various hole offsets of the LSR membrane were then tested to evaluate their performance.

### 2.4. COMSOL Simulation

In all the simulations, solid mechanics models in COMSOL were utilized. Specifically, contact pairs were defined between the membrane and the via, as these components come into contact during assembly without penetrating each other. The plastic material used was polycarbonate, while the membrane consisted of a silicone rubber sheet. The mechanical properties of both materials were sourced from the manufacturers’ product specifications.

## 3. Results and Discussion

### 3.1. Valves Without Pre-Stretching

For valve set A without membrane pre-stretch, different water column heights were used to evaluate whether the valve could remain closed. Table 1 presents the test results. A 20 mm water column represents the static pressure of a sample inside a microfluidic device, 100 mm simulates the pressure caused by gentle shaking, and 200 mm emulates the impact of a gentle bump, all of which mimic typical scenarios encountered during the practical use of medical devices in laboratories. As shown in Table 1, low via heights (below 0.448 mm) fail to provide reliable valve closure. While higher via heights may occasionally close the valve at low pressure, they are inconsistent. During assembly, friction between the compression beads and the membrane can randomly stretch the membrane slightly, which explains why the valve sometimes closes at low pressure. However, at 200 mm (~1.96 kPa) water column pressure, none of the tested via heights achieved a normally closed valve. These findings indicate that simply laying down an elastomer membrane over a raised via and clamping its edges is insufficient to construct a reliable normally closed valve.

**Figure 4:**
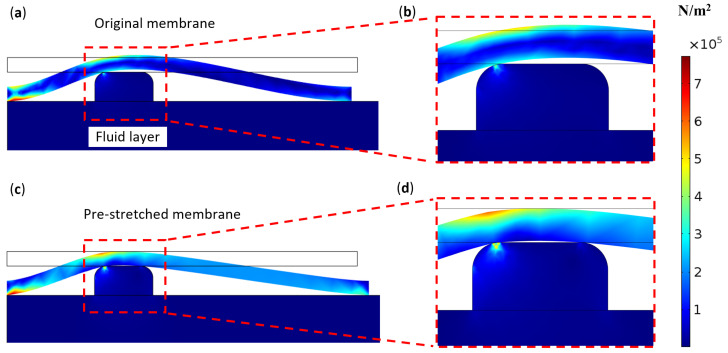
COMSOL simulation results showing membrane deformation after being laid over the via and clamped at the edges. (**a**) Membrane deformation without pre-stretch. (**b**) Zoomed-in view of the membrane deformation from (**a**). (**c**) Membrane deformation with 0.2 mm pre-stretch. (**d**) Zoomed-in view of the membrane deformation from (**c**).

COMSOL simulation results further illustrate the deformation of the membrane on the via and demonstrate its inability to seal the fluid via effectively. In this simulation, a 40A durometer elastomer was modeled to contact a 1 mm diameter via and was then pressed down onto the surface of the fluid layer on both sides. The left side of the membrane was fixed onto the fluid layer surface in both the vertical and horizontal directions, while the right side was constrained with zero prescribed displacement in the vertical direction only, mimicking the pick-and-place membrane assembly process. As shown in Figure 4a, the membrane curved downward and made contact with the via. However, because the left side was pinned to the fluid layer, the right side of the membrane shifted inward slightly to compensate for the membrane’s curved profile. Although the membrane contacted the via, it failed to establish a tight seal around it. Figure 4b provides a zoomed-in view of the via, showing that the membrane does not touch the via on the right side, leaving it partially open. This opening clearly prevents the membrane from forming a proper seal for fluids. In practice, the size of this via opening may vary due to manufacturing tolerances. If the opening is small enough, surface tension may prevent fluid flow through the gap, explaining why the valve closed in some low-pressure cases observed in the experiments shown in Table 1. However, these results indicate that this approach cannot produce a reliable normally closed valve, as corroborated by the experimental data in the same table.

### 3.2. Valves with Pre-Stretching

The proposed membrane pre-stretching method effectively addresses this issue, as demonstrated by the COMSOL simulation results in Figure 4c,d. In this simulation, the left edge of the membrane was pinned to the surface of the fluidic layer in both the vertical and horizontal directions, while the right edge was pre-stretched by 0.2 mm before being pressed down. As shown in Figure 4d, this pre-stretch allowed the membrane to completely seal the via. The level of pre-stretch can be adjusted to accommodate different sealing requirements for various applications, as exemplified by valve set B in this study.

**Figure 5:**
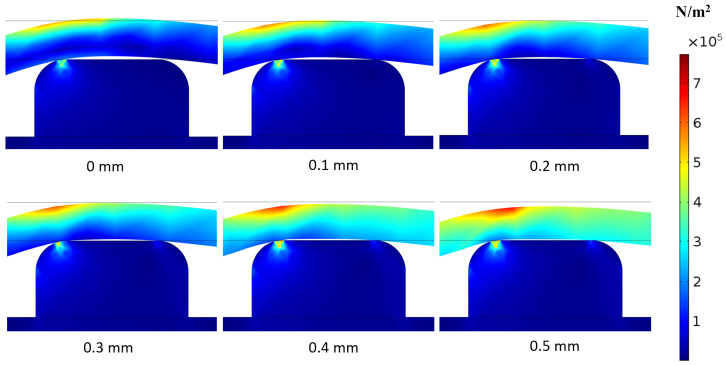
Simulation results showing membrane deformation and stress on the via edges with various pre-stretch levels, ranging from 0 to 0.5 mm. The via diameter is 1 mm, included for scale reference.

For valve set B with various pre-stretch levels, COMSOL simulations were performed for each offset (Figure 5). The results indicate that at 0 offset, the valve remains open. When the offset is increased to 0.1 mm, the membrane comes into contact with the via, thereby closing the valve through VMP. As the offset increases (0 mm, 0.1 mm, 0.2 mm, 0.3 mm, 0.4 mm, and 0.5 mm), the VMP exerted by the membrane on the via also increases, as indicated by the color legend. The simulation results show VMP values for each offset of 0 Pa, 12,108 Pa, 68,760 Pa, 139,870 Pa, 168,642 Pa, and 199,230 Pa, respectively. This demonstrates that increasing the offset results in higher pressure on the via, providing a stronger seal and ensuring the valve remains reliably closed.

Experiments were conducted with these different offsets using the setup shown in Figure 3. Briefly, water columns of 20 mm (196 Pa), 100 mm (980 Pa), and 200 mm (1961 Pa) in height were connected to the fluidic channel to test whether the head pressure of the water column would open the valve. The results are presented in Table 2. Except for one valve with a 0.1 mm offset failing at the 200 mm water column, all valves with offsets greater than 0.1 mm functioned as normally closed valves. Notably, at the 0.1 mm offset, although the contact pressure on the via from the simulation was 12,108 Pa, which is significantly higher than the pressure generated by a 200 mm water column, one out of three tests still failed. This failure could be attributed to manufacturing defects or imperfections in the via surface finish. These findings highlight that even when an offset theoretically provides a strong seal for a normally closed valve, practical factors such as manufacturing and assembly tolerances must be considered. Selecting a greater offset can help mitigate these risks and enhance the reliability of the normally closed valve.

**Figure 6:**
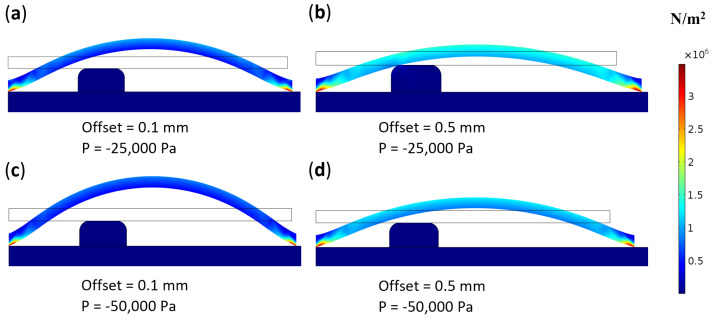
Effect of varying vacuum strengths on pre-stretched membranes at different levels. The via diameter is 1 mm, included for scale reference.

A functional normally closed valve should not only remain closed under normal use conditions but also open successfully when required. Valve opening is achieved by applying negative air pressure to the top of the membrane in the pneumatic layer, as shown in Figure 1a. When the membrane is pre-stretched, the in-plane stress increases its rigidity, making it more difficult to lift under negative pressure. To verify whether valves with pre-stretched membranes could open successfully, we performed a simulation in which negative pressures of −25,000 Pa and −50,000 Pa were applied to the top of membranes with 0.1 mm and 0.5 mm pre-stretch. The simulation results indicated that both pressure levels could successfully open the valve for both pre-stretch levels. At greater pre-stretch, the opening size is smaller (Figure 6b); however, applying stronger vacuum pressure can lift the membrane further, creating a larger opening (Figure 6d). To ensure reliable valve opening and smooth fluid flow, selecting a stronger vacuum pump may be necessary.

Experiments were conducted to verify whether the selected vacuum pump (KNF NMP830KPDC) could successfully open the valves at different pre-stretch levels, using the setup shown in Figure 3c. The vacuum was supplied to the cavity on top of the membrane via the yellow needle. The vacuum pump can generate a maximum negative pressure of −75,000 Pa (~11 psi). A 20 mm water column was connected to one end of the fluid channel, and fluid flow from one end to the other through the via was observed to confirm that the valve had opened. The test results demonstrated that all valves, across all pre-stretch levels (0.1 mm, 0.2 mm, 0.3 mm, 0.4 mm, and 0.5 mm), opened successfully.

### 3.3. Normally Closed Valve Application in a Molecular Diagnostic Assay

**Figure 7:**
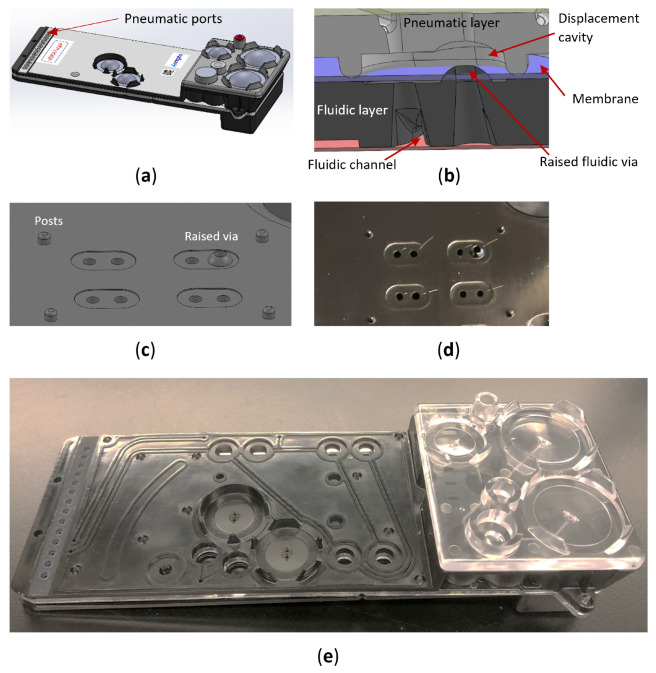
A robust NCV design for an IVD application. (**a**) Microfluidic cartridge design with pneumatic ports on the top. (**b**) Cross-sectional view of the normally closed valve. (**c**) Valve design showing posts used to stretch the membrane. (**d**) Injection-molded plastic fluidic layer of the microfluidic cartridge. (**e**) Partial assembly of the cartridge showing all the plastic layers of the cartridge.

After verifying this concept, we implemented it in a lab-on-a-chip cartridge designed for a liquid biopsy diagnostic application. This cartridge incorporates DNA extraction, purification, and DNA amplification and detection functions in one plastic chip (Figure 7a). Once the sample is loaded into the cartridge, the user can walk away and wait for actionable results in 90 min. The cartridge was designed using SolidWorks and fabricated through injection molding. Figure 7a illustrates the overall cartridge design. A row of pneumatic manifolds on the left side interfaces with the pressure supply on the instrument, providing either positive or negative pressure for fluid flow control. Due to the large volume (~10 mL) of the sample mix in the cartridge (located on the top right), fluids can flow uncontrollably in the microfluidic channel under gravity, potentially disrupting the sample and reagent manipulation. To address this issue and ensure precise control of the sample, a normally closed valve (NCV) was incorporated. Figure 7b shows the cross-sectional details of the NCV design. Four posts are used to stretch the membrane (Figure 7c) by 0.5 mm, ensuring a reliable seal on the via. Figure 7d displays the details of the valves design on the injection-molded polycarbonate components of the fluidic layer and Figure 7e the partially assembled cartridges for engineering testing. The entire cartridge was assembled using a combination of laser welding and heat staking.

The raised height of the fluidic via is *h* = 0.5 mm, and the distance between the posts, or the width of the membrane, is *w* = 16 mm. The silicone elastomer membrane, with a thickness of 0.25 mm and a Shore A hardness of 40, was purchased from Stock-well Elastomeric (Philadelphia, PA, USA). The membrane was laser-cut using a Boss LS1416 (Sanford, FL, USA) into the required shapes and assembled into the cartridge by stretching it onto the posts on the fluidic layer. The plastic components were then laser-welded using a Leister system (Itasca, IL, USA) to clamp the membrane securely with compression beads on the pneumatic layer, forming the normally closed valve.

The assembled cartridge and the NCV were tested using in-house developed instruments. Once the cartridge was loaded into the instrument, a pneumatic manifold connected to a vacuum pump (KNF NMP830KPDC, Trenton, NJ, USA) engaged with the pneumatic ports on the cartridge to supply the desired pressure for controlling the valve or fluid flow inside the cartridge. A total of 8 mL of fluid was loaded into the sample container on the cartridge to test the NCV. This product was prototyped, and hundreds of cartridges were tested using engineered samples, with the NCV performing repeatedly and reliably; no NCV failure was observed with 8 mL of sample loading. Building on the successful valve design and cartridge functionality, clinical sample testing is now in progress.

## 4. Conclusions

We proposed and constructed a simple and robust normally closed valve for microfluidic cartridge flow control. By pre-stretching and clamping an LSR membrane onto a raised microfluidic via, a contact pressure is generated on the via to prevent fluids from flowing uncontrollably. When a negative pressure is applied from above, the membrane lifts, opening the valve and allowing fluid to flow in a controlled manner. Both numerical analysis and experiments were conducted to verify this concept. The numerical analysis provided guidance on membrane selection, pre-stretch determination, and vacuum pump selection, while the experimental results highlighted practical factors to consider, especially manufacturing and assembly tolerances. Both numerical and experimental results demonstrate that the proposed normally closed valve operates repeatedly and reliably for lab-on-a-chip flow control applications, where precise pneumatic fluid flow control is crucial.

## Figures and Tables

**Table 1:** Valve closing performance without membrane pre-stretch.

Via Heights (mm)	Water Column Heights (mm)
	(# of Successfully Closed/# of Tests)
	20 100 200
0.381	0/3 0/3 0/3
0.406	0/3 0/3 0/3
0.432	0/3 0/3 0/3
0.448	1/3 1/3 0/3
0.477	1/3 0/3 0/3
0.508	2/3 0/3 0/3

For each via height, three repeats were performed on three individual samples to include the fabrication variation.

**Table 2:** Valve closing performance with various membrane pre-stretches; the via height is 0.508 mm.

Pre-Stretch (mm)	Water Column Heights (mm)
	(# of Successfully Closed/# of Tests)
	20 100 200
0.1	3/3 3/3 2/3
0.2	3/3 3/3 3/3
0.3	3/3 3/3 3/3
0.4	3/3 3/3 3/3
0.5	3/3 3/3 3/3
0.6	3/3 3/3 3/3

For each pre-stretch, three repeats were performed on three individual samples to include the fabrication variation.

## Data Availability

The raw data supporting the conclusions of this article will be made available by the authors on request.
